# Induction chemotherapy followed by concurrent chemoradiotherapy versus concurrent chemoradiotherapy alone in stage III-IVb nasopharyngeal carcinoma patients with Epstein-Barr virus DNA ≥4000 copies/ml: a matched study

**DOI:** 10.18632/oncotarget.8828

**Published:** 2016-04-18

**Authors:** Shan-Shan Guo, Lin-Quan Tang, Qiu-Yan Chen, Lu Zhang, Li-Ting Liu, Ling Guo, Hao-Yuan Mo, Dong-Hua Luo, Pei-Yu Huang, Yan-Qun Xiang, Rui Sun, Ming-Yuan Chen, Lin Wang, Xing Lv, Chong Zhao, Xiang Guo, Ka-Jia Cao, Chao-Nan Qian, Mu-Shen Zeng, Jin-Xin Bei, Ming-Huang Hong, Jian-Yong Shao, Ying Sun, Jun Ma, Hai-Qiang Mai

**Affiliations:** ^1^ Sun Yat-sen University Cancer Center, State Key Laboratory of Oncology in South China, Collaborative Innovation Center for Cancer Medicine, Guangzhou 510060, China; ^2^ Department of Nasopharyngeal Carcinoma, Sun Yat-sen University Cancer Center, Guangzhou 510060, China; ^3^ Good Clinical Practice center, Sun Yat-sen University Cancer Center, Guangzhou 510060, China; ^4^ Department of Molecular Diagnostics, Sun Yat-sen University Cancer Center, Guangzhou 510060, China; ^5^ Department of Radiation Oncology, Sun Yat-sen University Cancer Center, Guangzhou 510060, China

**Keywords:** nasopharyngeal carcinoma, induction chemotherapy, concurrent chemotherapy, IMRT, EBV DNA

## Abstract

**Background:**

The effects of induction chemotherapy (IC) followed by concurrent chemoradiotherapy (CCRT) in high-risk (stage III-IVb with EBV DNA≥4000 copies/ml) nasopharyngeal carcinoma (NPC) patients are unclear.

**Methods:**

A total of 325 high-risk NPC patients treated with IC+CCRT or CCRT alone who were treated with intensity-modulated radiation therapy (IMRT) between March 2007 and March 2013 were included. For each patient in the IC+CCRT group, a matched pair in the CCRT group was matching for: gender, age, T stage, N stage, clinical stage and WHO (World Health Organization) type. The primary endpoint was overall survival (OS), and the secondary endpoints were progression-free survival (PFS), distant metastasis-free survival (DMFS), and locoregional relapse-free survival (LRFS).

**Results:**

There were no significant differences in OS, PFS, DMFS, and LRFS between the IC+CCRT (148 patients) and CCRT (177 patients) groups. After matching, 103 paired patients were analyzed, and there were no differences between the IC+CCRT and CCRT groups regarding clinical outcomes. Based on the subgroup analysis of 156 very-high-risk patients (stage N2-3 with EBV DNA ≥4000 copies/ml), the 5-year OS of the IC+CCRT and CCRT groups was 84.3% and 67.5% (P =0.033), respectively. Based on our multivariate analysis, the treatment group was significantly associated with OS (P=0.034; HR0.451, 95%CI 0.216-0.941).

**Conclusions:**

IC+CCRT did not improve the clinical outcomes of high-risk NPC patients compared to CCRT alone. However, in very-high-risk patients, IC+CCRT treatment led to increased OS compared to patients received CCRT treatment alone.

## INTRODUCTION

Nasopharyngeal carcinoma (NPC) is epidemic in Southern China and Southeast Asia but is rare worldwide. The incidence of NPC in Southern China is approximately 15–30 for every 100,000 people per year [1]. NPC differs from other head and neck carcinomas in its correlation with the Epstein–Barr virus (EBV), aggressive natural locoregional history, greater tendency for distant metastases and special therapeutic considerations [2, 3]. Radiotherapy is the primary treatment modality, and for locoregionally advanced NPC, concurrent chemoradiotherapy (CCRT) is recommended as the standard of care based on large-scale clinical trials and meta-analysis [4–9]. With the widespread use of intensity modulated radiotherapy (IMRT), a local control rate of up to 90% has been achieved for locally advanced NPC [10–12]. Presently, distant metastases are the primary cause of treatment failure. A phase III study in Taiwan comparing CCRT with radiotherapy alone showed that CCRT improved survival rates of low-risk patients, in the meantime, reduced local recurrence rate and distant metastasis rate [9]. However, no significant results were observed for high-risk patients. Induction chemotherapy (IC) has been considered to show good compliance and has the ability to shrink tumor size and eliminate micrometastasis. A randomized phase II trial in Hong Kong comparing docetaxel and cisplatin IC+CCRT with CCRT alone in advanced NPC showed that patients receiving IC+CCRT had improved OS [13]. However, the 3-year progression-free survival (PFS) rate and OS rate were very similar between the IC+CCRT and CCRT groups in a randomized phase II study conducted by the Hellenic Cooperative Oncology Group (HeCOG) that compared IC+CCRT with weekly cisplatin versus the same CCRT in cases of locally advanced NPC [14]. Recently, a randomized phase II/III trial of locally advanced NPC in Singapore showed no differences in survival between induction chemotherapy with GCP (gemcitabine, carboplatin, and paclitaxel) prior to CCRT and CCRT alone in cases of locally advanced NPC [15]. Because of these inconsistent results of clinical trials, there has been controversy regarding the effects of induction chemotherapy on NPC, suggesting that the selection of an appropriate target patient group could be important. MAC meta-analysis has revealed that while induction chemotherapy did not improve OS, it decreased distant metastasis free survival (DMFS) rate and improved local control rate [4]. EBV deoxyribonucleic acid (DNA) is an important prognostic factor for NPC patients, and EBV DNA combined with TNM staging could identify patients at high risk of locoregional recurrence and distant metastasis within a group of locoregionally advanced NPC patients [16]. Previous studies of stratified NPC patients have identified an EBV DNA concentration of 4000 copies/ml as a prognostic cut-off value [17–19]. Induction chemotherapy might be combined with CCRT to improve the treatment outcomes in cases of high-risk NPC. Therefore, it is necessary to evaluate the role of induction chemotherapy in high-risk, locoregionally advanced NPC patients receiving concurrent CCRT based on IMRT.

In this study, we compared the clinical outcomes in patients with locoregionally advanced NPC with a pretreatment EBV DNA concentration of ≥ 4000 copies/ml treated with cisplatin plus fluorouracil induction chemotherapy regimen followed by CCRT to those receiving CCRT alone.

## RESULTS

### Patient characteristics

Table 1 shows the baseline clinical characteristics of patients before and after matching. We analyzed a total of 325 consecutive locoregionally advanced NPC patients who were treated with CCRT followed by PF induction chemotherapy (IC) regimen and patients treated with cisplatin CCRT between March 2007 and March 2013 at Sun Yat-sen University Cancer Center. A total of 206 patients were included in this study after matching, and the two groups were well balanced due to their accordance with the strict matching conditions. The ratio of males to females in each group was 2.68:1, with a total of 75 males and 28 females. The median follow-up time after matching was 49 months (range: 9-100) and 50 months (range: 4-100) for the IC+CCRT and CCRT groups, respectively.

**Table 1:** Baseline characteristics of patients with stage III-IVb nasopharyngeal carcinoma

	Unmatched			Matched		
	IC+CCRT	CCRT	*P*-value	IC+CCRT	CCRT	*P*-value
	148(45.5%)	177(54.5%)		103(50.0%)	103(50.0%)	
**Age (yr),**			0.175			0.887
<45	73(49.3%)	74(41.8%)		40(38.8%)	41(39.8%)	
≥45	75(50.7%)	103(58.2%)		63(61.2%)	62(60.2%)	
**Gender**			0.351			1.000
Female	48(32.4%)	49(27.7%)		28(27.2%)	28(27.2%)	
Male	100(67.6%)	128(72.3%)		75(72.8%)	75(72.8%)	
**T stage**			0.322			1.000
1	1(0.7%)	5(2.8%)		0(0.0%)	0(0.0%)	
2	24(16.2%)	28(15.8%)		19(8.8%)	19(18.4%)	
3	71(48.0%)	94(53.1%)		53(57.1%)	53(57.6%)	
4	52(35.1%)	50(28.2%)		31(34.1%)	31(30.1%)	
**N stage**			0.001			1.000
0	17(11.5%)	8(4.5%)		5(4.9%)	5(4.9%)	
1	22(14.9%)	42(23.7%)		20(19.4%)	20(19.4%)	
2	78(52.7%)	110(62.1%)		67(65.0%)	67(65.0%)	
3	31(20.9%)	17(9.6%)		11(10.7%)	11(10.7%)	
**Clinical stage**			<0.001			0.399
**III**	70(47.3%)	114(64.4%)		62(60.2%)	64(62.1%)	
**IVa**	50(33.8%)	54(30.5%)		31(30.1%)	34(33.0%)	
**IVb**	28(18.9%)	9(5.1%)		10(9.7%)	5(4.9%)	
**WHO type**			0.650			0.316
**1**	0(0.0%)	1(0.6%)		0(0%)	0(0%)	
**2**	3(2.0%)	4(2.3%)		0(0%)	1(1.0%)	
**3**	145(98.0%)	172(97.2%)		103(100%)	102(99.0%)	

Abbreviations: IC =induction chemotherapy; CCRT= concurrent chemoradiotherapy.

### Survival outcomes before matching

In the unmatched population (n = 325), 49 patients (15.1%) had died, 13 patients (4.0%) had locoregional recurrence, and 49 patients (15.1%) had distant metastasis. The 5-year OS, 5-year PFS, 5-year LRFS and 5-year DMFS rates were 88.1%, 86.4%, 96.4% and 88.2%, respectively. The median duration of follow-up for the unmatched population was 52.4 (range: 2-100) months.

The 5-year OS rates for the IC+CCRT (148 patients) and CCRT groups (177 patients) were 87.9% (95%CI 82.2-93.6) and 81.1% (95%CI 74.8-87.4), respectively (P=0.088). The 5-year PFS rates for the IC+CCRT and CCRT groups were 78.6% (95%CI 70.8-86.4) and 82.4% (95%CI 76.1-88.7), respectively (P=0.558). The 5-year LRFS rates for the IC+CCRT (95%CI 88.9-97.9) and CCRT groups were 93.4% and 96.3% (95% CI 93.0-99.6), respectively (P=0.248). The 5-year DMFS rates for the IC+CCRT and CCRT groups were 81.5% (95%CI 74.1-88.9) and 85.0% (95%CI 79.3-90.7), respectively (P=0.787). There were no differences in the OS, PFS, LRFS and DMFS rates between the IC+CCRT and CCRT groups.

We analyzed other potential prognostic factors in the overall population before matching, and we found that BMI (≤24 or >24), family history, age, gender, clinical stage, WHO type, ECOG score, ACE-27 score, smoking status, and T stage (T1-2, T3-4) were not associated with the OS, PFS, LRFS and DMFS rates. Patients at the N2-3 stage had poorer OS rates compared to N0-1 patients (P=0.031). In our multivariate analysis, the N stage and treatment modalities (CCRT vs. IC+CCRT) were independent prognostic factors for OS among the high-risk patients, with HRs of 1.90 (P=0.031, 95%CI 1.061-3.405) and 0.472 (P=0.022, 95%CI 0.248-0.899), respectively.

### Survival outcomes after matching

**Figure 1:**
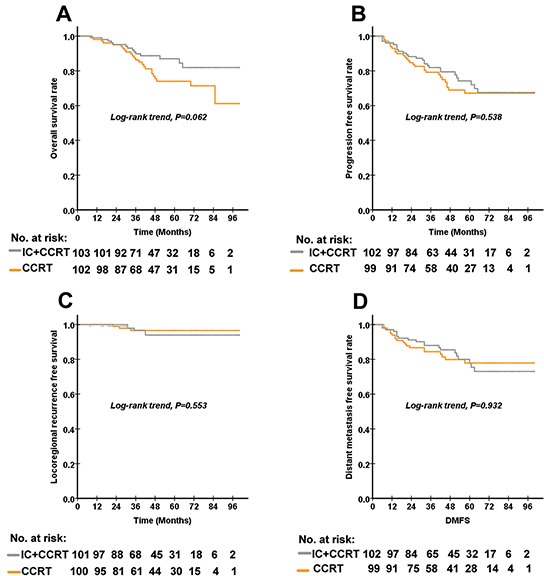
The Kaplan–Meier curves for OS, PFS, LRFS and DMFS according to the treatment arm in 103 paired (a total of 206) patients with high-risk nasopharyngeal carcinoma

The matching process led to a balanced study population consisting of IC+CCRT (n = 103) and CCRT (n = 103) groups. The median duration of follow-up for the 206 patient-matched population was 50.0 (range: 4-100) months. Figure 1 shows the Kaplan–Meier curves for the OS, PFS, LRFS and DMFS rates of the patients after matching. The 5-year OS rates for the IC+CCRT and CCRT groups were 87.0% (95%CI 73.6-87.8) and 74.0% (95%CI 64.4-83.6), respectively (P=0.062). The 5-year PFS rates for the IC+CCRT and CCRT groups were 74.2% (95%CI 64.8-83.6) and 67.1% (95%CI 56.7-77.5), respectively (P=0.538). The 5-year LRFS rates for the IC+CCRT and CCRT groups were 93.9% (95%CI 88.6-99.2) and 96.6% (95%CI 92.9-100.0), respectively (P=0.553). The 5-year DMFS rates for the IC+CCRT and CCRT groups were 77.7% (95%CI 76.7-78.7) and 77.9% (95%CI 68.7-87.1), respectively (P=0.932). There were no differences in the OS, PFS, LRFS and DMFS rates between the IC+CCRT and CCRT groups. In our multivariate analysis, the N stage was an independent prognostic factor for OS, with an HR of 2.377 (P=0.015, 95%CI 1.187-4.759).

**Figure 2:**
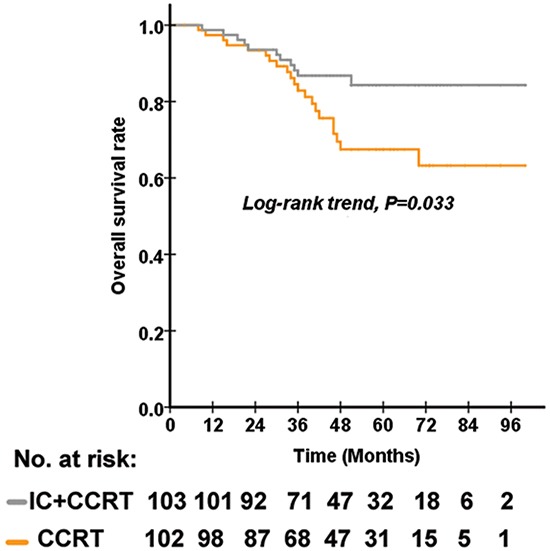
The Kaplan–Meier curves of OS according to the treatment arm from 156 very-high-risk NPC patients

As the N stage was an independent prognostic factor for OS and previous studies have used N2 as a cut-off value in the analysis of subgroup patients [17, 20], we defined patients of N2-3 stage with an EBV DNA ≥4000 copies/ml as being very high risk. The number of patients and other characteristics for the subgroup analysis of the 156 very-high-risk patients were shown in Table 2. For our subgroup analysis of 156 very-high-risk patients (N2-3 stage with EBV DNA ≥4000 copies/ml), the OS rate was significantly associated with inclusion in either the IC+CCRT or CCRT treatment group. The 5-year OS rates for the IC+CCRT and CCRT groups were 84.3% (95%CI 75.5%-93.1%) and 67.5% (95%CI 55.3%-79.7%), respectively (P=0.033) (Figure 2). The 5-year DMFS rates for stage N2-3 and stage N0-1 were 74.5% (95%CI 66.3%-82.7%) and 87.2% (95%CI 0.762-0.982), respectively (P=0.062). The 5-year PFS rates for the IC+CCRT and CCRT groups were 67.6% (55.8%-79.4%) and 60.2% (95%CI 47.7-72.7%), respectively (P=0.419). The 5-year DMFS rates for the IC+CCRT and CCRT groups were 71.1% (95%CI 59.3%-82.9%) and 64.2% (95%CI 51.9%-76.5%), respectively (P=0.389). The 5-year LRFS rates for the IC+CCRT and CCRT groups were 93.5% (95%CI 87.2%-99.8%) and 96.8% (95%CI 92.5-101.1%), respectively (P=0.523). In our multivariate analysis using Cox proportional regression model, the treatment group was significantly associated with OS (P=0.034), and the patients who received IC+CCRT had a lower risk of death compared to those who received CCRT alone, with an HR of 0.451 (95%CI 0.216-0.941) (Table 3).

**Table 2:** Baseline characteristics of 156 very high-risk subgroup patients (stage N2-3 with EBV DNA ≥4000 copies/ml)

	IC+CCRT	CCRT	*P*-value
	79(50.0%)	77(50.0%)	
Age (yr)			0.888
<45	34(43.0%)	34(44.2%)	
≥45	45(57.0%)	43(55.8%)	
Gender			0.928
Female	19(24.1%)	19(24.7%)	
Male	60(75.9%)	58(75.3%)	
T stage			0.877
2	18(22.8%)	20(26.0%)	
3	46(58.2%)	42(54.5%)	
4	15(19.0%)	15(19.5%)	
N stage			0.948
2	68(86.1%)	66(85.7%)	
3	11(13.9%)	11(14.3%)	
Clinical stage			0.384
III	54(68.4%)	54(70.1%)	
IVa	15(19.0%)	18(23.4%)	
IVb	10(12.7%)	5(6.5%)	
WHO type			0.759
1	0	0	
2	0	0	
3	79(100.0%)	77(100.0%)	

**Table 3:** Multivariate analysis of 156 N2-3 stage patients with EBV DNA≥4000 copies/ml

Characteristics	OR	95%CI	*P* value
Age	1.381	0.664-2.874	0.387
Gender	0.509	0.23-1.161	0.109
T stage	1.178	0.672-2.067	0.567
Clinical stage	1.559	0.907-2.679	0.108
Treatment arm	0.451	0.216-0.941	0.034

Abbreviations: EBV DNA= Epstein-Barr virus deoxyribonucleic acid; OR=odds ratio.

## DISCUSSION

Until now, EBV DNA has been the most effective predictive biomarker for guiding the treatment of NPC. NPC patients with high pre-treatment levels of EBV DNA had a higher risk for disease recurrence and distant metastasis [18, 21]. For such high-risk NPC patients, a more intensive treatment regimen such as induction chemotherapy in addition to CCRT could be promising because induction chemotherapy is tolerated better than adjuvant chemotherapy and has the ability to decrease local recurrence and distant metastasis. However, there are no studies demonstrating the effects of induction chemotherapy combined with CCRT in high-risk patients with high pre-treatment EBV DNA levels. To our knowledge, this is the first study in the IMRT era to directly compare the effects of IC+CCRT to CCRT alone.

Although there is no improvement in OS among the high-risk patients receiving IC+CCRT, we found that very-high-risk patients (i.e., stage N2-3 with EBV DNA≥4000 copies/ml) who received the PF IC+CCRT regimen had an improved 5-year OS compared to those who underwent treatment with CCRT alone.

This result suggests that among locally advanced NPC patients, those representing very high risk might gain a long-term benefit in terms of OS. A subgroup analysis of 284 locally advanced NPC patients in Taiwan concluded that CCRT has advantages over RT alone in low-risk patients but showed no benefit in high-risk patients [22], indicating that high-risk patients might require more intensive chemotherapy. Our study suggests a promising means to screen patients who are at very high risk and thus appropriate for treatment with more powerful combined chemoradiotherapy. Additional prospective randomized studies comparing IC+CCRT with CCRT alone in stage N2-3 patients with EBV DNA≥4000 copies/ml are needed to validate this finding.

We did not observe significant differences in DMFS between the IC+CCRT and CCRT groups for the high-risk patients or the very-high-risk patients. Our results showed that patients receiving IC+CCRT had improved OS rates compared to those receiving CCRT. The difference in OS between the CCRT and IC+CCRT groups could be explained by the results of joint effects on distant metastasis and locoregional recurrence. Within the entire cohort, 42 cases died of distant metastasis, 5 cases died of tumor relapse, 1 patient died of cardiovascular disease, and 1 died for unknown reasons; most of the patients died of distant metastasis. Although we did not observe differences in DMFS between the IC+CCRT and CCRT groups in the high-risk patients or in the very-high-risk patients, we found that patients receiving IC+CCRT had a trend toward increased DMFS, PFS and LRFS times relative to the CCRT group (Supplementary Table S1). We speculate that the prolonged DMFS time together with the LRFS time resulted in a significantly prolonged OS time in the IC+CCRT group compared to the CCRT group. Although the DMFS did not differ between the IC+CCRT and CCRT groups, the combination of effects on DMFS and LRFS led to the significant improvement in OS.

Among all of the high-risk locoregional advanced NPC patients with EBV DNA levels ≥4000 copies/ml treated with IMRT, the present study did not find any benefit of the PF induction chemotherapy regimen compared to CCRT alone. The results suggest that adding other effective anti-tumor chemotherapy agents to induction chemotherapy could improve the prognosis of NPC. For example, adding docetaxel to cisplatin and fluorouracil induction chemotherapy plus CCRT provided a long-term survival benefit to patients with head and neck cancers compared to the PF IC+CCRT regimen [23, 24]. Whether the addition of docetaxel to cisplatin and fluorouracil induction chemotherapy would provide a long-term survival benefit in locally advanced NPC patients requires further study. Sun Yat-sen University is studying the effects of the induction TPF regimen followed by concurrent cisplatin plus RT compared to RT plus concurrent cisplatin without induction in stage III-IVB disease (NCT01245959). The unpublished data from this phase III trial are expected to clarify the effects of TPF induction chemotherapy plus CCRT. It is necessary to find a superior method for combining chemotherapy with RT in high-risk patients.

There are some limitations to this study. First, our study is retrospective, and although we use a method of matching to decrease potential bias, there is inevitable bias caused by its retrospective nature. Further prospective studies are needed to validate our results. Second, this is a single-center analysis from a high-prevalence district. A multi-center study is needed to fully evaluate the effects of IC+CCRT on high-risk NPC patients.

In conclusion, our study found that IC+CCRT did not improve clinical outcomes in high-risk NPC patients receiving IMRT compared with CCRT alone. However, in very-high-risk patients (i.e., stage N2-3 with EBV DNA≥4000 copies/ml), IC+CCRT treatment leads to increased OS compared to those patients who underwent treatment with CCRT alone. Future prospective studies are needed to further validate our results.

## MATERIALS AND METHODS

### Patient recruitment

**Figure 3:**
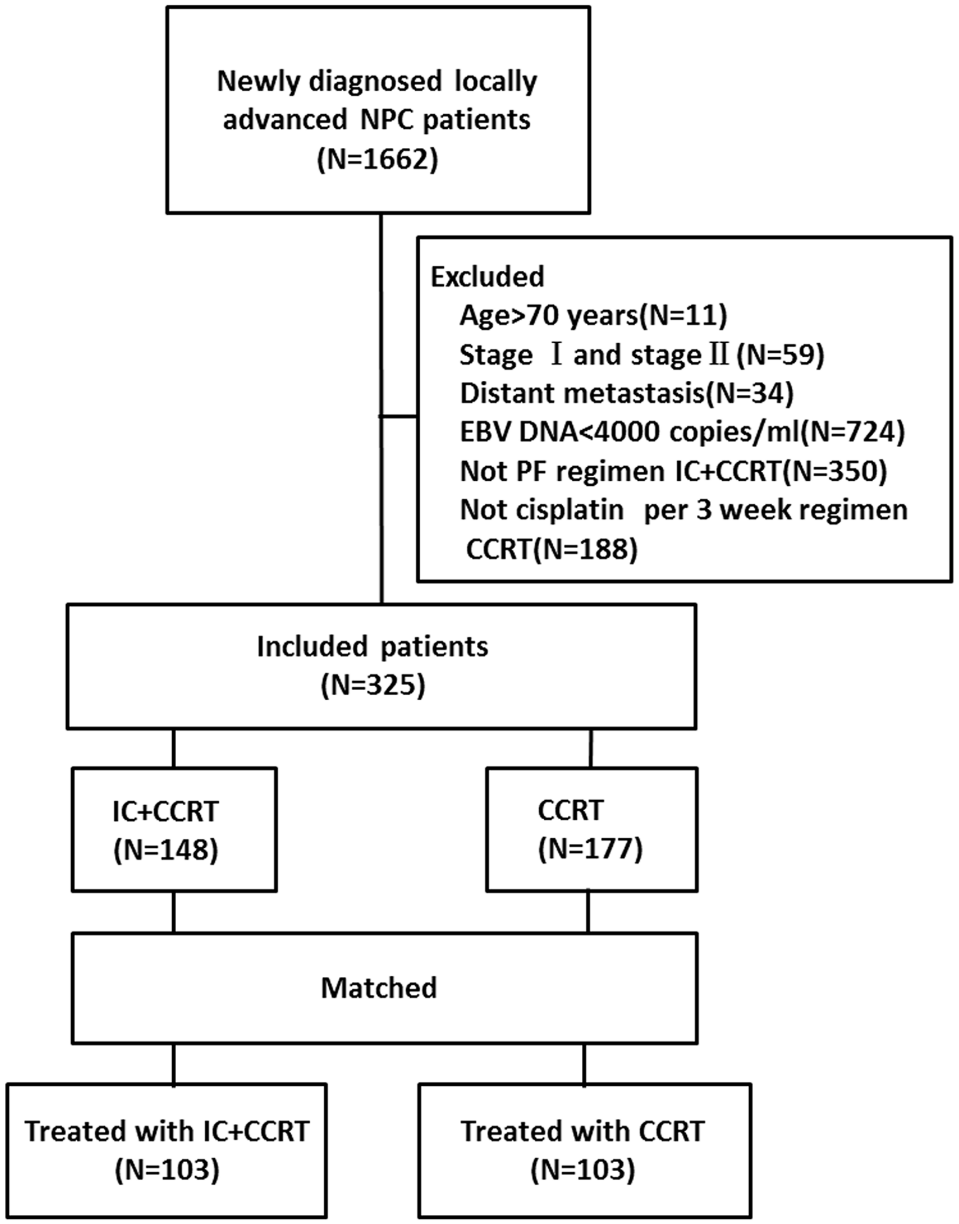
Study flow diagram

In this retrospective study, we reviewed a total of 1662 consecutive NPC patients who had received IMRT. Ultimately, 325 patients with histologically confirmed locoregionally advanced NPC (stage III-IVb with EBV DNA≥4000 copies/ml) who had been treated with CCRT followed by induction chemotherapy and patients treated with CCRT alone between March 2007 and March 2013 at Sun Yat-sen University Cancer Center were included (Figure 3). All of the included patients had plasma EBV DNA levels of ≥4000 copies/ml prior to treatment. The following criteria were met by this patient population: NPC confirmed histologically by a biopsy of the nasopharynx; no distant metastasis; no treatment prior to admission; no other tumor types or serious illnesses; an Eastern Cooperative Oncology Group (ECOG) performance score ≤2; plasma EBV DNA≥4000 copies/ml before treatment; and treatment with radical IMRT. Patients who were more than 70 years old; were stage I or stage II; had distant metastasis; had not received PF IC+CCRT regimen; had not received cisplatin per the 3-week CCRT regimen; or whose plasma EBV DNA levels were <4000 copies/ml before treatment were excluded. The staging workup included an MRI of the head and neck, a chest radiograph, a bone scintigraphy, and an ultrasonography of the abdominal region for all patients. All of the included patients were restaged according to the Seventh Edition of the American Joint Committee on Cancer (AJCC) staging system. After matching, 103 paired (a total of 206) patients were analyzed further. Table 1 shows the clinicopathological features in the study population of un-matched and matched patients.

### Plasma EBV DNA assay

The plasma EBV DNA concentrations of patients were measured by quantitative polymerase chain reaction prior to treatment as described in previous studies [25, 26]. The cut-off value of 4000 copies/ml was based on previous studies [16, 17].

### Treatment

The target volumes were delineated according to a previously described institutional treatment protocol [27], which is in accordance with the International Commission on Radiation Units and Measurements reports 50 and 62. All of the target volumes were depicted slice-by-slice on the treatment planning computed tomography scan. The primary nasopharyngeal gross tumor volume (GTVnx) and the involved cervical lymph nodes were determined based on the imaging, clinical, and endoscopic findings. The enlarged retropharyngeal nodes together with primary gross tumor volume (GTV) were outlined as the GTVnx on the IMRT plans. The first clinical tumor volume (CTV1) was defined as the area from 0.5-1.0 cm outside the GTV, a site that involves potential sites of local infiltration. Clinical target volume 2 (CTV2) was defined as the margin from 0.5-1.0 cm around CTV1 and the lymph node draining area (Levels II, III, and IV). For stage N1–3 patients, the lower neck area received conventional anterior cervical field radiation in daily fractions of 2 Gy with a midline shield to 50 Gy. For patients with stage N0 disease, RT was not delivered to the lower neck area. The prescribed dose was 66–70 Gy to the planning target volume (PTV), 60 Gy to PTV1, 54 Gy to PTV2, and 60–66 Gy to PTV of the involved cervical lymph nodes in 30 to 33 fractions. In total, 30–33 fractions were administered at 1 fraction per day, 5 days/week. The IMRT plan was designed in accordance with previous studies conducted at the Sun Yat-sen University Cancer Center [28, 29].

Induction chemotherapy was composed of fluorouracil (3000-3500 mg/m^2^ given on days 1-5 via continuous intravenous infusion for 120 hours) and cisplatin (80 mg/m^2^) for 2-3 courses. Concurrent chemotherapy was initiated on the first day during IMRT, and cisplatin was delivered by an intravenous infusion (IV) of 80–100 mg/m^2^ every 3 weeks for 2-3 courses.

The primary endpoint was overall survival (OS), and secondary endpoints were progression-free survival (PFS), distant metastasis-free survival (DMFS), and locoregional relapse-free survival (LRFS). The OS was defined as the time from NPC treatment to death from any cause or until the date of the last follow-up. PFS was defined as the time from the NPC treatment to events that included death or disease progression at local, regional, or distant sites or until the date of the last follow-up. LRFS was defined as the time from the NPC treatment to the absence of primary site or neck lymph node relapse or until the date of the last follow-up. DMFS was defined as the time from the NPC treatment to the date of the first observation of distant metastases or until the date of the last follow-up.

### Follow-up

All patients were followed at regular intervals after radiotherapy, which were every 2 months during the first 6 months, every 3 months for the next 6 months, every 4 months during the second year, and every 6 months thereafter. The median follow-up period for the entire patient cohort was 51.3 months (range 2–100 months). There were 11 cases lost during follow-up, and the follow-up rate was 96.6%.

### Statistical analysis

The differences between the means of continuous variables were compared using Student's t-test. Categorical variables were compared using Pearson's x^2^ test or Fischer's exact test. Survival rates were calculated using the Kaplan–Meier method, and prognostic factors and survival curves were compared using the log-rank test. Multivariate analyses were calculated using the Cox proportional hazards regression model. The potentially important prognostic factors considered in the matching process included the following: gender (female, male), age (<45, ≥45), T stage (T1, T2, T3, T4), N stage (N0, N1, N2, N3), clinical stage (III, IV a, IV b) and WHO type (I, II, III).

For each patient in the IC+CCRT group, a matched pair in the CCRT group was identified by matching for gender (female, male), age (<45, ≥45), T stage (T1, T2, T3, T4), N stage (N0, N1, N2, N3), clinical stage (III, IV a, IV b) and WHO type (I, II, III); we matched 1:1 pairs of IC+CCRT and CCRT patients. The two matched subgroups were then analyzed for OS, PFS, LRFS and DMFS by univariate and multivariate analyses. All *P* values were two-tailed; P ≤0.05 was considered statistically significant. The program Statistical Package for Social Sciences version 18 (SPSS Inc., Chicago, IL, USA) was used for analysis.

## SUPPLEMENTARY TABLE


