# Supplementary material for: Induction chemotherapy followed by concurrent chemoradiotherapy versus concurrent chemoradiotherapy alone in stage III-IVb nasopharyngeal carcinoma patients with Epstein-Barr virus DNA ≥4000 copies/ml: a matched study
Source: Oncotarget. 2016 Apr 18;7(20):29739–48. doi: 10.18632/oncotarget.8828 (PMC5045429; doi:10.18632/oncotarget.8828)
Supplement: Supplementary file 1 [file oncotarget-07-29739-s001.pdf]

SUPPLEMENTARY TABLE

Supplementary Table S1: The comparison of DMFS, LRFS, and PFS between the CCRT and IC+CCRT groups

	CCRT	IC+CCRT	*P* value
**DMFS (Median, Months)**	46	49	0.219
**LRFS (Median, Months)**	48	50	0.319
**PFS (Median, Months)**	45	48	0.12

Abbreviations: DMFS=distant metastasis-free survival; LRFS=locoregional recurrence-free survival; PFS=progression-free survival.
